# Influence of *Schistosoma japonicum* programmed cell death protein 10 on the growth and development of schistosomula

**DOI:** 10.1186/s13071-018-2636-8

**Published:** 2018-01-18

**Authors:** Yan Ru Gao, Wen Ling Huang, Chun Lian Tang, Rong Liu, Qin Ping Zhao, Zhen Ping Ming, Hui Fen Dong

**Affiliations:** 1 Hubei Province Key Laboratory of Allergy and Immunology, Department of Parasitology, Wuhan University School of Basic Medical Sciences, Wuhan, Hubei Province 430071 China; 2 Department of Physiology, School of Basic Medicine Sciences, Hubei University of Science and Technology, Xianning, Hubei Province 437000 China; 3 Medical Department, City College, Wuhan University of Science and Technology, Wuhan, Hubei Province 430083 China; 4 Department of Clinical Laboratory, Wuchang Hospital, Wuhan, 430063 China

**Keywords:** *Schistosoma japonicum*, Schistosomula, Programmed cell death factor-10, Growth and development, Tegument, Apoptosis

## Abstract

**Background:**

Schistosomiasis caused by *Schistosoma japonicum* is among the most serious endemic zoonoses in China. To study interactions between schistosomula, the pre-adult juvenile stage, and hosts, it is important to study the functions of key genes involved in schistosomula growth and development. Programmed cell death protein 10 (*pcdp*10) is an important apoptosis-related gene with various biological functions. This study described the molecular characterization of *S. japonicum* PCDP10 (*Sj*PCDP10) and evaluated its functions in schistosomula.

**Methods:**

Real-time quantitative polymerase chain reaction (qPCR) and western blot were used to detect *Sjpcdp*10 mRNA and protein levels, respectively, at different developmental stages. Immunolocalization was performed to determine *Sj*PCDP10 expression in the parasite. RNA interference (RNAi) experiments were used to assess gene functions associated with *Sj*PCDP10 in schistosomula growth and development.

**Results:**

Real-time qPCR revealed that *Sjpcdp*10 was expressed during all investigated developmental stages and upregulated during schistosomula growth and development. Histochemical localization showed that *Sj*PCDP10 was mainly distributed in the teguments of schistosomula in all investigated stages and part of the parenchymal area of 14-, 18-, and 21-day-old schistosomula. Following *Sjpcdp*10 knockdown by RNAi, the lengths, widths, areas, and volumes of schistosomula were significantly lower than those in the control group. Scanning electron microscopy showed that the body surfaces of schistosomula subjected to RNAi were seriously damaged, with few tegumental spines and sensory papillae. Transmission electron microscopy indicated that the teguments of *Sjpcdp*10-knockdown schistosomula were incomplete, the number of layers was reduced, and the thickness decreased significantly as compared with those in the control group. Furthermore, terminal deoxynucleotidyl transferase dUTP nick-end labelling results showed that the rate of apoptosis in *Sjpcdp*10-knockdown schistosomula was significantly higher than that in the control group.

**Conclusions:**

*Sjpcdp*10-knockdown influenced the growth and development of schistosomula. Therefore, our results indicated that *Sj*PCDP10 contributes to the regulation of cell apoptosis and is essential for schistosomula growth and development.

**Electronic supplementary material:**

The online version of this article (10.1186/s13071-018-2636-8) contains supplementary material, which is available to authorized users.

## Background

Schistosomiasis is a “neglected tropical disease” that affects over 200 million people in 78 countries and is considered a chronic and poverty-promoting disease [1]. *Schistosoma japonicum* is the only human blood fluke that occurs in China. As of 2015, there were > 30, 000 villages (containing 68 million residents) endemic for schistosomiasis [2]. As one of the countries routinely affected by epidemic diseases, China has made great strides in schistosomiasis. However, there are emerging challenges, including limitations in new drug development and the lack of an available vaccine [3–6]. Although some vaccines have been developed, the ideal immunity rate has not been achieved [5]. One reason for this might be that growth and developmental mechanisms and its interactions with the host are poorly understood [7]. To this end, the study of schistosomulum-specific molecules is not only important for identifying new functional genes as potential vaccine antigens or drug targets for human schistosomiasis but also helpful in revealing mechanisms of growth, development, and interactions with the host. Therefore, it is necessary to study the functions of the key genes involved in *S. japonicum* growth and development in more detail.

Studies of schistosomulum staging reported that inhibiting the expression of cathepsin B in *Schistosoma mansoni* might lead to schistosomula growth retardation [8]. Furthermore, RNAi-mediated knockdown of the 26S proteasome subunit SmRPN11/POH1 affects schistosomula development and survival [9]. Our laboratory screened for genes, such as orthologues of *adenylate kinase* 1*, programmed cell death protein* 10 (*pcdp*10), and *heat-shock protein* 70, that might play an important role in the growth and development of *S. japonicum* schistosomula [10, 11]. Among these, PCDP10 is an important molecule involved in apoptosis regulation and was originally named TF-1 cell apoptosis-related factor-15 based on its cloning from human erythroleukemia TF-1 cell lines [12]. Functional studies have shown that PCDP10 exhibits a variety of biological functions in human cells, including inhibiting tumour-cell apoptosis and promoting blood-vessel regeneration and reconstruction [13, 14]. Although there are many reports regarding the function of human PCDP10, few focused on parasite PCDP10 proteins, including that of *S. japonicum* (*Sj*PCDP10). *Sjpcdp*10 was also found to be differentially expressed between schistosomula cultured in vitro and those derived from hosts [10], and it was hypothesized that *Sj*PCDP10 might be involved in schistosomula growth and development. Therefore, in this study, the sequence characteristics, localization, and expression levels of *Sj*PCDP10 were analyzed throughout the life-cycle of *S. japonicum*. Our results showed that RNA-mediated *Sjpcdp*10 knockdown resulted in severe morphological damage. These results deepen our understanding of the biological role of this gene in *S. japonicum* development.

## Methods

### Animals and parasites

Female Kunming mice (6–8 week-old, 20–25 g each) were purchased from Wuhan University Laboratory Animal Centre and randomly divided into six groups. Two adult male New Zealand rabbits (2.5 kg each) and *Oncomelania hupensis* snails infected with *S. japonicum* were purchased from Hubei Provincial Center for Disease Control and Prevention. Cercariae were collected routinely by exposing *O. hupensis* snails in water to light for 3 to 4 h to induce parasite shedding. Sixty mice were divided into six groups and infected with different numbers of cercariae (8000, 5000, 800, 600, 200 and 100 cercariae) via abdominal-skin exposure. After infection, mice were killed and the schistosomulae were collected at different time points.

(30 min, 3 days, 10 days, 14 days, 18 days and 21 days after infection). For skin-type schistosomula (30 min), infected skin was directly removed, cut into pieces and then incubated with 10 ml phosphate-buffered saline (PBS) at 37 °C for 2 h. The incubated mixture was filtered through a filter screen (140-mesh) (Wuhan Kerui Biological Technology Co. Ltd., Wuhan, China) [10]. The other schistosomula were collected by perfusion of mice according to methods previously described [15]. All experimental data were derived from in vivo living schistosomula except the schistosomulae of RNAi experiments, which was mechanically transformed by cercariae in vitro.

### Molecular cloning and sequence analysis of *Sj*PCDP10

Full-length complementary DNA (cDNA) was obtained using reverse transcription polymerase chain reaction (RT-PCR) with mRNA template prepared from lung-stage schistosomula (3 days after infection). Primers were designed by Oligo 6 software (http://www.oligo.net/) per the *Sjpcdp*10 mRNA sequence (accession no. FN326945.1) obtained from GenBank. Forward (5′-CCG GAA TTC ATG GCT GGA AGT AAG TGG C-3′) and reverse (5′-CCG CTC GAG ATC CAC ATC GTG AAC C-3′) primers containing *EcoR*I and *Xho*I restriction sites were used to amplify the target gene by PCR according the following amplification protocol: 95 °C for 5 min; 35 cycles at 95 °C for 30 s, 58 °C for 30 s, and 72 °C for 90 s, followed by a final extension at 72 °C for 8 min. The PCR products were purified using AxyPrep DNA gel extraction kit (Axygen, Hangzhou, China) according to the standard protocol and cloned into the prokaryotic expression vector pET28a (+) (Novagen, Madison, USA). Recombinant *Sjpcdp*10 (r*Sjpcdp*10) plasmids were transformed into competent *Escherichia coli* DH5α cells, and positive clones were screened and identified by PCR, enzyme analysis, and sequencing (Sunny Biotechnology Co., Ltd., Shanghai, China).

The molecular characteristics of *Sj*PCDP10 were analyzed using a variety of bioinformatics approaches. The amino acid sequence was used as a query to identify PCDP10 orthologues. An alignment of protein sequences exhibiting sufficient similarity from different species was generated by ClusalX 2.0 (http://www.clustal.org/clustal2/). A phylogenetic tree was generated using the neighbour-joining method by MEGA5.05 software (The Biodesign Institute, Tempe, AZ, USA) to analyze relationships between *Sj*PCDP10 and PCDP10 homologs from other species. The stability of the amino acid sequences was predicted by ProtParam (http://web.expasy.org/protparam/).

### RNA extraction and analysis of *Sjpcdp*10 mRNA expression by real-time quantitative PCR (qPCR)

Total RNA was extracted from schistosomula using TRIzol reagent (Invitrogen, Carlsbad, CA, USA), according to manufacturer instructions. After removing the genomic DNA with RNase-free DNase (Takara Bio, Shiga, Japan), cDNA was synthesized using the RevertAid First Strand cDNA synthesis kit (Fermentas, Vilnius, Lithuania), according to the standard protocol. All reactions were performed with a Bio-Rad CFX96 detection system (Bio-Rad, Hercules, CA, USA). Primers used for real-time qPCR were designed with Beacon Designer version 8.14 software (PREMIER Biosoft, Palo Alto, CA, USA) and synthesized by Sunny Co., Ltd. Primer sequences are shown in Additional file 1: Table S1. Primers were used to amplify a 142-bp fragment of *Sjpcdp*10 (GenBank: FN326945.1), and another pair of primers was used for amplifying a 213-bp fragment of the *α-tubulin* gene of *S. japonicum* (GenBank: AY815746.1) as an internal control [16]. Melting curve analyses of the specific PCR products were performed, and each experiment was performed in triplicate. Bio-Rad CFX Manager 3.1 software (Bio-Rad) was used to analyze *Sjpcdp*10 transcript levels relative to those of *α-tubulin*, according to the 2^-△△Ct^ method [17].

### Immunolocalization of r*Sj*PCDP10

The recombinant pET28a (+)-*Sj*PCDP10 plasmids were prepared using the AxyPrep plasmid miniprep kit (Axygen, Hangzhou, China) and then transformed into competent *E. coli* BL21 (DE3) cells (Novagen, Madison, USA) to express the r*Sj*PCDP10 protein. A large amount of r*Sj*PCDP10 protein was expressed at 37 °C for 3 h following induction with 0.5 mM isopropyl-β-thiogalactopyranoside and purified using Ni-NTA agarose (QIAGEN, Hilden, Germany) affinity purification according to manufacturer instructions. Purified protein was quantified using the BCA protein assay kit (Beyotime, Shanghai, China) according to manufacturer instructions and administered to New Zealand male rabbits in the neck and back by multipoint subcutaneous injection at a dose of 1 mg/kg r*Sj*PCDP10 emulsified with adjuvant. The immunization was performed three times at time intervals of 2 weeks. Ear venous blood was exsanguinated from the rabbits and used to assess the antibody titer by enzyme-linked immunosorbent assay. Anti-r*Sj*PCDP10 sera were collected 2 weeks after the final immunization. Negative control sera were collected from rabbits immunized with 8 M urea emulsified with an adjuvant.

All parasites were fixed with 4% paraformaldehyde overnight at room temperature (20–25 °C) and then embedded in paraffin. Immunolocalization of *Sj*PCDP10 was assessed by an immunohistochemical method previously described [18]. Rabbit-anti-r*Sj*PCDP10 serum (1:100 dilution; sera prepared as described) was used as the primary antibody, and Cy3-conjugated goat anti-rabbit (red fluorescence) was used as the secondary antibody (1:3000 dilution; KPL, Gaithersburg, MD, USA). Cell nuclei were stained blue fluorescence using 4′,6-diamidino-2-phenylindole dye solution for 5 min and washed with PBS. After fixing with a cover glass containing anti-fade mounting medium (Beyotime), slides were observed under a fluorescence microscope (OLYMPUS, Tokyo, Japan), followed by semi-quantitative analysis by measuring the optical density value of *Sj*PCDP10 protein distribution area using IPP6.0 software (Media Cybernetics, Rockville, USA).

### Western blot analysis

Total soluble protein was obtained from worms at different stages according to a method described previously [19]. Purified r*Sj*PCDP10 (4 μg) and total soluble protein (20 μg) of schistosomula were subjected to sodium dodecyl sulfate-polyacrylamide gel electrophoresis and then transferred electrophoretically onto a 0.45-μm pore nitrocellulose membrane (Whatman; GE Healthcare, Little Chalfont, UK) at 80 V for 70 min. Membranes were blocked with 5% (*w*/*v*) non-fat dried milk in tris-buffered saline with 0.05% (*v*/v) Tween-20 (TBS/T) for 1 h at 37 °C. Membranes were then incubated overnight with anti-r*Sj*PCDP10 rabbit serum (1: 1000) and anti-α-tubulin rabbit polyclonal antibody (1:3000 dilution; TDYBIO, Beijing, China) at 4 °C. After washing with TBS/T every 5 min for four rounds, membranes were incubated with horseradish peroxidase-conjugated goat anti-rabbit secondary antibody (1:5000 dilution; KPL) for 2 h at 37 °C. The membranes were then washed with TBS/T every 5 min for three rounds and visualized using enhanced chemiluminescence reagent (ASPEN, Wuhan, China), according to manufacturer instruction.

### RNA-mediated *Sjpcdp*10 knockdown

*Sjpcdp*10-specific dsRNA and irrelevant *enhanced green fluorescent protein* (*egfp*) dsRNA (negative control) were synthesized in vitro with the T7 RiboMAX expression RNAi system (Promega, Durham, NC, USA) according to manufacturer instructions. Primers tagged with T7 RNA polymerase promoter sequences at both ends (Additional file 2: Table S2) were designed with the help of the online IDT RNAi Design Tool (https://sg.idtdna.com/site/order/designtool/index/DSIRNA_CUSTOM) to amplify a 560-bp DNA product from *Sjpcdp*10 cDNA by PCR. Primers for *egfp* were the same as reported by Liu et al. [18] and were used to amplify a 678-bp DNA product from pEGFP-N1 plasmids by PCR. dsRNA was prepared by DNA transcription in vitro and stored at -20 °C after purification.

Cercariae were collected as described, mechanically transformed into schistosomula by syringe passage under sterile conditions [10], and cultured in vitro with “841” medium [a mixture of RPMI1640 (Gibco; Thermo Fisher Scientific, Waltham, MA, USA), 10% fetal bovine serum (Gibco), 1 μM 5-hydroxytryptamine, 1 μM L-hydrocortisone, 0.5 μM hypoxanthine, 0.2 U/ml insulin, 100 U/ml penicillin, 100 μg/ml streptomycin [10], and a concentration gradient of 0.5, 1, 2, 4, 8, 16, 32 or 64 × 10^−5^ mg/ml *Sjpcdp*10 dsRNA, with no dsRNA as a blank control and 4 × 10^−5^ mg/mL *egfp* dsRNA (same as the optimal concentration of *Sjpcdp*10 dsRNA) used as a negative control. Schistosomula were cultured at 37 °C in an incubator with 5% CO_2_ for 1 to 7 days, with the medium and dsRNA replaced every other day.

To monitor gene expression and dsRNA concentrations at various time points following dsRNA soaking, real-time qPCR was performed using the conditions described. To measure protein levels after RNAi, western blot analysis was performed after RNAi under optimal conditions. To compare the parasite sizes of the control and RNAi groups, images were obtained under a stereomicroscope (Olympus SZX7; OLYMPUS), and the lengths (L) and widths (W) of schistosomula were analyzed by Image-pro plus 6.0 software (Media Cybernetics), followed by L: W ratio calculations [10]. Routine dehydration, desiccation and spray gold were performed, and SEM was used to observe the surface morphology using a VEGA 3 LMU scanning electron microscope (TESCAN, Brno, Czech Republic). TEM specimens were fixed, dehydrated, embedded and then cut into ultrathin sections. Ultrastructural alterations were observed by H-7700 transmission electron microscopy (TEM; Hitachi, Tokyo, Japan). The terminal deoxynucleotidyl transferase dUTP nick-end labelling (TUNEL) method (in situ cell death detection kit; Roche, Basel, Sweden) was used to examine the occurrence of apoptosis in schistosomula after *Sjpcdp*10 knockdown, and images were obtained under an IX51 inverted microscope (OLYMPUS).

### Statistical analysis

All data are expressed as the mean ± standard deviation. Statistical analysis for quantitative RT-PCR and antibodies was performed by analysis of variance. A *P <* 0.05 was considered significant. All RNAi results were compared with those of the blank control group.

## Results

### Molecular cloning and sequence analysis of *Sj*PCDP10

*Sjpcdp*10 cDNA (GenBank: FN326945.1) contains an open reading frame of 651-bp, encoding a putative 216-aa protein with a predicted molecular weight of 26 kDa and a calculated isoelectric point of 9.11. Bioinformatics analysis revealed that *Sj*PCDP10 is an unstable (instability index: 40.46) and hydrophilic (GRAVY: -0.269) protein. No typical signal-peptide sequence, a transmembrane region, or N-glycosylation sites were predicted according to SignalP 4.1 (http://www.cbs.dtu.dk/services/SignalP/), TMHMM version. 2.0 (http://www.cbs.dtu.dk/services/TMHMM/), and NetNGlyc 1.0 (www.cbs.dtu.dk/services/NetNGlyc/).

**Fig. 1:**
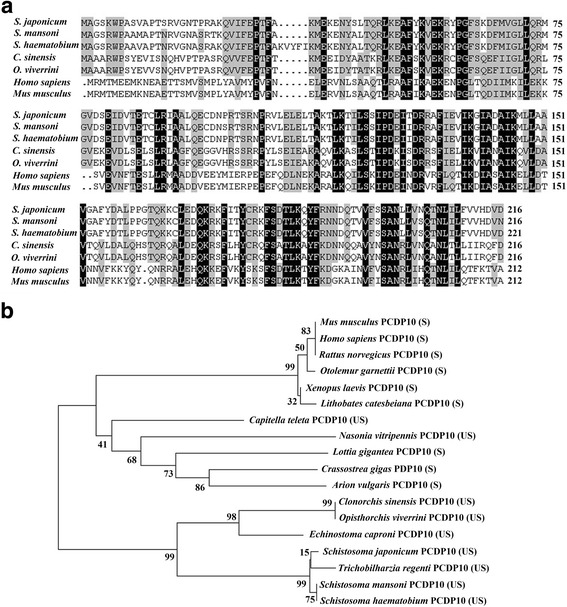
Bioinformatics analysis of *Schistosoma japonicum* PCDP10 (*Sj*PCDP10). **a** Amino acid sequence alignment of PCDP10 from *S. japonicum*. Black background indicates 100% identity in all species, dark grey indicates ≥75% identity, and light grey indicates ≥50% identity. **b** Phylogenetic tree of *Sj*PCDP10 and homologs from other species generated using the neighbour-joining method. The *Sj*PCDP10 protein was most closely related to PCDP10 proteins of *Schistosoma mansoni* and *Schistosoma haematobium* and showed low homology with those from other species. UniProtKB accession numbers are as follows: *S. japonicum* (Q5D8L4), *S. mansoni* (C4Q5Z1), *S. haematobium* (A0A095APY2), *Clonorchis sinensis* (H2KTN8), *Opisthorchis viverrini* (A0A074ZX12), *Trichobilharzia regent* (A0A183X0F3), *Echinostoma caproni* (A0A183AMZ2), *Capitella teleta* (R7TWK6), *Caenorhabditis elegans* (Q17958), *Homo sapiens* (Q9BUL8), *Mus musculus* (Q8VE70), *Rattus norvegicus* (Q6NX65), *Otolemur garnettii* (H0XA12), *Lithobates catesbeiana* (C1C3N3), *Crassostrea gigas* (K1PTV5), *Strigamia maritima* (T1JN96), *Nasonia vitripennis* (K7IPU5), *Arion vulgaris* (A0A0B6Y0C7), *Lottia gigantea* (V4B5U4), *Zootermopsis nevadensis* (A0A067R7A2), and *Xenopus laevis* (Q8AVR4). *Abbreviations*: S, stable; US, unstable

ClustalX 2.0 alignment of the *Sj*PCDP10 sequence with PCDP10 homologs from trematodes and other species revealed that *Sj*PCDP10 shared the highest similarity with PCDP10 proteins from *Schistosoma mansoni* (96%) and *Schistosoma haematobium* (93%), followed by lower levels of similarity with those from *Clonorchis sinensis* (53%), *Opisthorchis viverrini* (53%), *Homo sapiens* (42%) and *Mus musculus* (42%) (Fig. 1a). Sequences of PCDP10 orthologues in species from several taxa were used to build a phylogenetic tree using the neighbour-joining method (Fig. 1b) [20]. *Sj*PCDP10 clustered with the PCDP10 proteins of trematodes (*S. mansoni*, *S. haematobium*, *C. sinensis*, *Echinostoma caproni*, *Trichobilharzia regenti* and *O. viverrini*), forming a common clade.

### *Sjpcdp*10 mRNA expression at different developmental stages

**Fig. 2:**
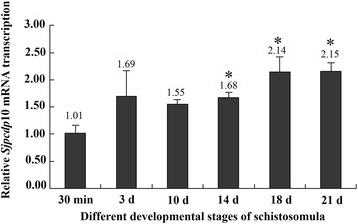
*Sjpcdp*10 transcript levels in the schistosomulum stage of the *Schistosoma japonicum* life-cycle. Stages included skin-stage schistosomula (30 min), 3-day-old lung-stage schistosomula (3 d), 10-day-old liver-stage schistosomula (10 d), 14-day-old liver-stage schistosomula (14 d), 18-day-old liver-stage schistosomula (18 d), and 21-day-old liver-stage schistosomula (21 d). *Sjpcdp*10 transcript levels at the different schistosomula stage were compared by multiple comparisons. The expression of *S. japonicum α-tubulin* was used as an internal control. All experiments were performed in triplicate, and the figure above each bar is the mean of each group. Asterisks denote significant differences (*P* < 0.05)

The transcript levels of *Sjpcdp*10 mRNA at different schistosomula stages were determined by real-time qPCR. As shown in Fig. 2, *Sjpcdp*10 mRNA was expressed at all investigated developmental stages. Compared with levels expressed in skin-stage schistosomula, the *Sjpcdp*10 expression levels were higher in 14-, 18-, and 21-day-old schistosomula (*F*_(5,12)_ = 8.69, *P* < 0.05). There was no significant difference among the other groups.

### r*Sj*PCDP10 immunolocalization

**Fig. 3:**
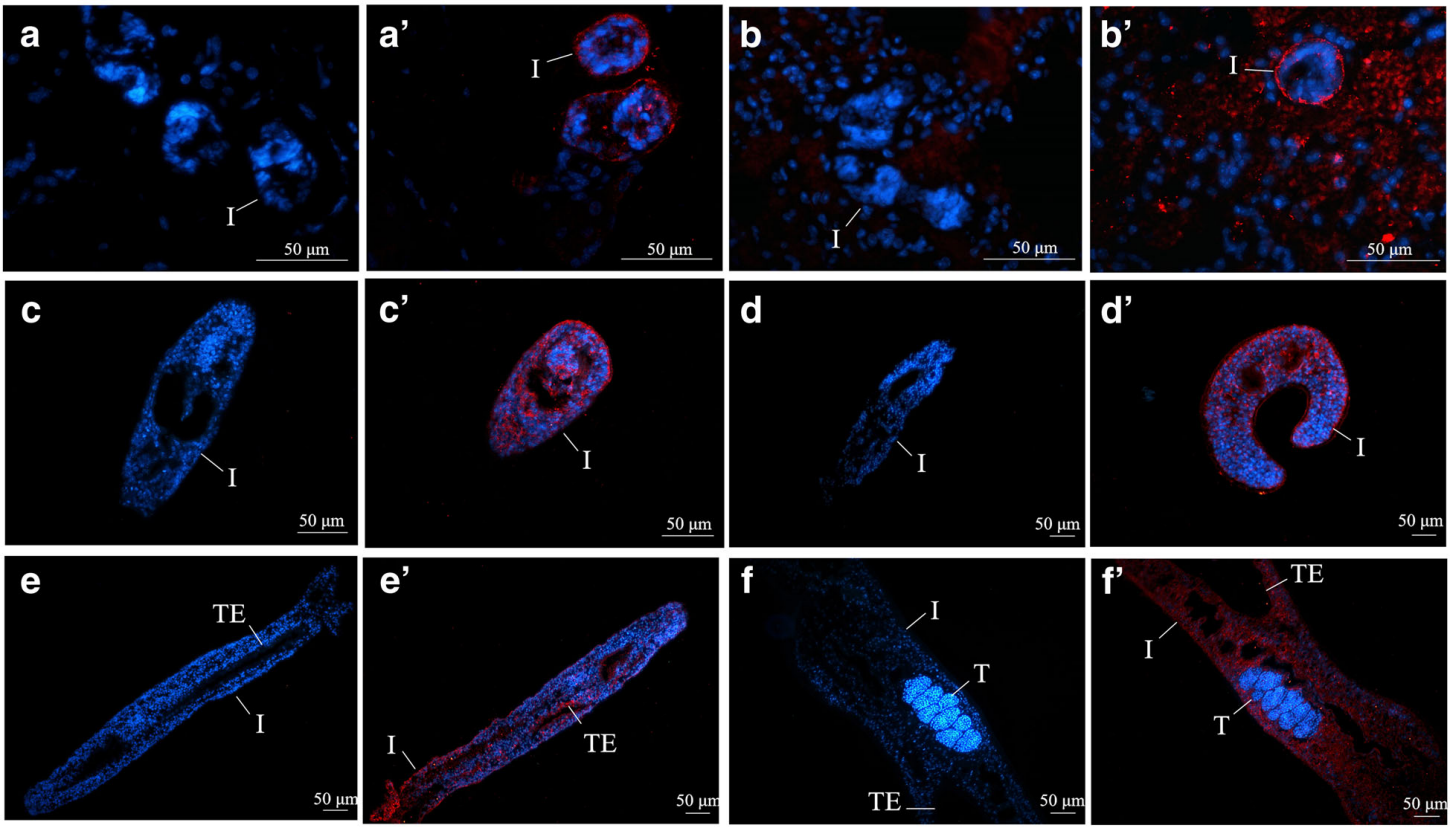
Immunolocalization of *Sj*PCDP10 in different tissues of *Schistosoma japonicum.* **a**-**f** Skin-stage, lung-stage, and 10-, 14-, 18-, and 21-day-old liver-stage schistosomula were probed with negative rabbit sera. **a’**-**f’** Skin-stage, lung-stage, and 10-, 14-, 18-, and 21-day-old liver-stage schistosomula were probed with anti-r*Sj*PCDP10 rabbit sera. *Sj*PCDP10 protein was found to be present in the different developmental stages of schistosomula. Red fluorescence indicates areas positive for the *Sj*PCDP10 protein. Blue fluorescence represents the cell nucleus. Images **a** and **a’** were obtained from the skin of mice infected with cercaria. Images **b** and **b’** were obtained from the lung of mice infected with cercaria. *Abbreviations*: I, integument; TE, intestinal epithelium; T, testis

An immunolocalization assay was performed to determine tissue localization of the *Sj*PCDP10 protein in schistosomula and using negative control serum as the primary antibody for the control group. As shown in Fig. 3, *Sj*PCDP10 was mainly distributed in the teguments of schistosomula in all investigated stages and part of the parenchymal areas of 14-, 18-, and 21-day-old schistosomula. The area of each positive region was calculated using IPP 6.0 software, revealing that *Sj*PCDP10 expression levels were lower in skin-stage and lung-stage schistosomula and higher in 21-day-old schistosomula (Additional file 3: Table S3), which was consistent with qPCR (Fig. 3) results and western blot result (Additional file 4: Figure S1).

### Preparation of dsRNA and analysis of optimal RNAi conditions by qPCR and western blot

**Fig. 4:**
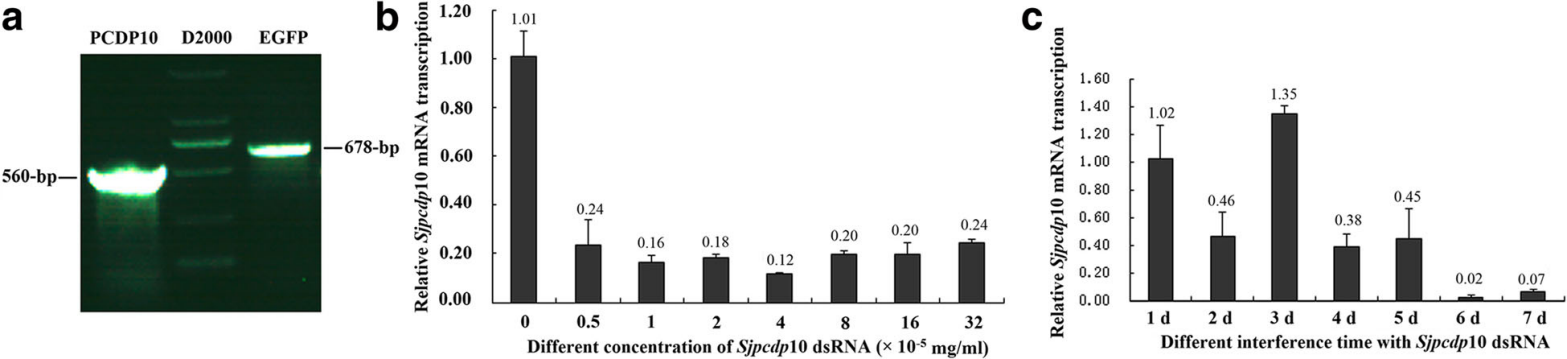
Preparation of *Sjpcdp*10 dsRNA and analysis of the optimal RNAi conditions. **a** Agarose gel electrophoresis of *Sjpcdp*10 dsRNA. Lane PCDP10: *Sjpcdp*10 dsRNA; Lane D2000: markers; Lane EGFP: *egfp* dsRNA (negative control dsRNA). **b** Analysis of the optimal concentration of *Sjpcdp*10 dsRNA at the optimal RNAi time (6 days). *Sjpcdp*10 dsRNA concentrations: 0, 0.5, 1, 2, 4, 8, 16, 32, 64 × 10^−5^ mg/ml. **c** Analysis of the optimal incubation/soaking time with the optimal concentration of *Sjpcdp*10 dsRNA (4 × 10^−5^ mg/ml). RNAi incubations times with *Sjpcdp*10 dsRNA: 1 d, 2 d, 3 d, 4 d, 5 d, 6 d, 7 d

dsRNA was synthesized using the *Sjpcdp*10 RNAi target sequence as a template and then added to the medium of schistosomula cultured in vitro to screen the optimal RNAi condition. RNAi efficiency was compared against the transcription level of *Sjpcdp*10 under different interference conditions. As shown in Fig. 4a, the size of *Sjpcdp*10 dsRNA was ~ 560-bp, which was consistent with the predicted result. *Sjpcdp*10 dsRNA was added to culture medium containing schistosomula cultured in vitro, followed by determination of the optimal RNAi conditions by qPCR. The optimal RNAi concentration of *Sjpcdp*10 dsRNA was 4 × 10^−5^ mg/ml, and the optimal RNAi duration was 6 days (Fig. 4b, c).

**Fig. 5:**
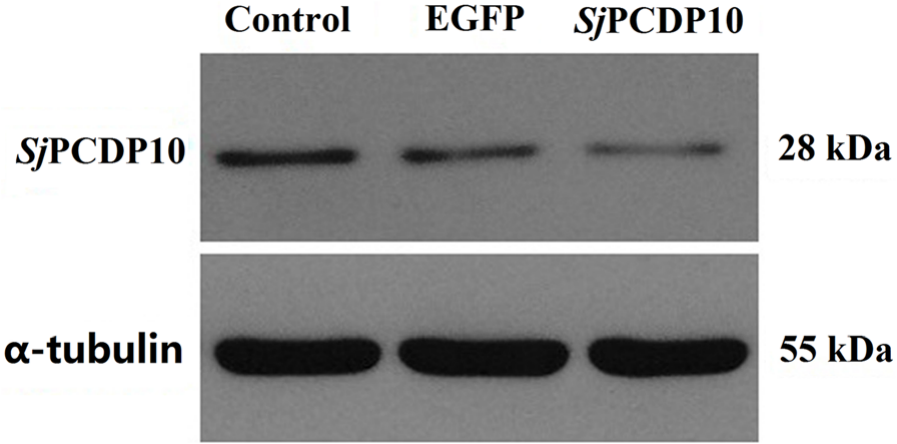
Western blot analysis of the effects of *Sjpcdp*10 knockdown. Lane 1: total protein of schistosomula from the control group; Lane 2: total protein of schistosomula from the *egfp* RNAi (negative control) group; Lane 3: total protein of schistosomula from the *Sjpcdp*10-knockdown group

dsRNA was added to the medium of schistosomula cultured in vitro at the optimal condition. Western blot results were analyzed using AlphaEaseFC software through the grey value ratio associated with *Sj*PCDP10: α-tubulin, which represented the relative *Sj*PCDP10 protein expression in different RNAi group. Compared with the control group (Fig. 5), *Sj*PCDP10 expression of the *Sjpcdp*10 dsRNA treated group decreased by 60% relative to that observed in the control group. Compared with the *egfp* negative control group, *Sj*PCDP10 expression decreased by 25%. These results showed that the RNAi experiment successfully achieved gene knockdown.

### Morphological changes in schistosomula after RNAi

**Fig. 6:**
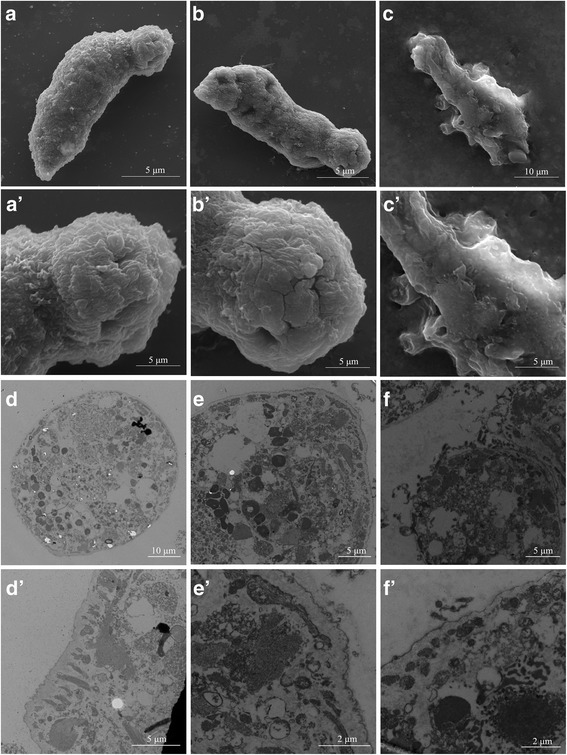
Ultrastructural changes in schistosomula after *Sjpcdp*10-knockdown experiments and analysis by electron microscopy. SEM analysis of morphological changes of the surface of blank control (**a**, **a’**), *egfp* negative control (**b**, **b’**) and *Sjpcdp*10-knockdown schistosomes (**c**, **c’**). TEM analysis of the morphological changes in the cell and tissue of blank control (**d**, **d’**), *egfp* negative control (**e**, **e’**) and *Sjpcdp*10-knockdown schistosomes (**f**, **f’**). SEM and TEM results of *Sjpcdp*10-knockdown schistosomes compared with those of the blank control and *egfp* negative control

The sizes of the schistosomula, and their ultrastructure was studied by SEM and TEM. The length, width, area, and volume of *Sjpcdp*10-knockdown schistosomula were significantly lower than those of the blank control group (Table 1). Based on SEM observations, schistosomula from the blank control (Fig. 6a, a’) or negative control (*egfp* RNAi) groups (Fig. 6b, b‘) both exhibited plump bodies with multiple spines distributed irregularly, as well as numerous sensory papillae on the body surface (Fig. 6a, a’, b, b’). By contrast, the surfaces of schistosomula subjected to *Sjpcdp*10 knockdown were severely damaged, with tegument structure profoundly disordered, fewer spines and no sensory papillae present at the surface (Fig. 6c, c‘). According to TEM, the tegument structures of schistosomula of the blank control (Fig. 6d, d‘) or negative control (*egfp* RNAi) groups (Fig. 6e, e‘) were intact, whereas the teguments of the *Sjpcdp*10-knockdown group were partially invaginated, interrupted, or bloated, and the subtegumental layer appeared reduced in thickness (Additional file 5: Figure S2) as compared with the two control group (Fig. 6f, f‘). Early apoptosis was observed in all groups according to the appearance of chromatin at the nuclear membrane and shrinkage of the nuclear membrane (Fig. 6f, f‘).

**Table 1:** Morphological changes of the schistosomula after interference

Group	RNAi time (days)	No. of worms	Mean length ± SD (μm)	Mean width ± SD (μm)	Mean volume ± SD (× 10^4^ μm^3^)	Mean area ± SD (× 10^4^ μm^2^)
Blank control	6	90	129.88 ± 17.10	66.19 ± 10.57	23.00 ± 7.72	3.41 ± 0.79
EGFP	6	77	127.31 ± 14.05	62.21 ± 8.59	20.63 ± 5.56	3.10 ± 0.58
*Sj*PCDP10	6	115	122.74 ± 14.20*	56.93 ± 6.45*	17.44 ± 4.03*	2.70 ± 0.41*

*P < 0.05

### Detection of apoptosis in schistosomula after RNAi by TUNEL assay

**Fig. 7:**
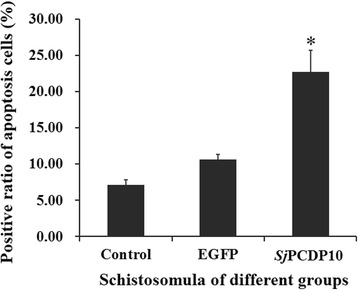
TUNEL assay analyzing schistosomula following *Sjpcdp*10 knockdown. Control: schistosomula from the blank control group; EGFP: schistosomula from the *egfp* negative control group; *Sj*PCDP10: schistosomula from the *Sjpcdp*10-knockdown group

Schistosomula were collected after RNAi, and paraffin sections were prepared. Rates of apoptosis were then determined by TUNEL assay, revealing that the rate of TUNEL-positive cells in the *Sjpcdp*10-knockdown group was 22.77% higher (*F*_(3,8)_ = 168.07, *P* < 0.05) than that of the control group (Fig. 7).

## Discussion

PCDP10 is an evolutionarily ancient, highly conserved gene with homologs in many different organisms, including nematodes*, Drosophila,* and zebrafish [14]*.* In this study, it was shown that *Sj*PCDP10 was highly similar to *S. mansoni* PCDP10 and *S. haematobium* PCDP10, with lower rates of similarity to other species, although a high degree of conservation was observed (Fig. 1a). The phylogenetic tree showed that the predicted PCDP10 proteins of trematodes and nematodes are unstable, whereas those of vertebrates are stable (Fig. 1b). Here, r*Sj*PCDP10 prepared using a prokaryotic expression system was unstable, consistent with the predicted results, and the purified r*Sj*PCDP10 protein was easily degraded into two small molecules of 15 kDa and 10 kDa. Therefore, r*Sj*PCDP10 must be freshly prepared before use in protein-immunization experiments. Currently, there are no reports concerning PCDP10 stability in other species, therefore, this requires additional data screening and experimental proof.

In humans, PCDP10 is widely expressed in various tissues, such as the placenta, liver, and kidney [12], as well as the uterus, ovary, lung, stomach, and small intestine, suggesting that PCDP10 might have a more generalized biological function [13]. In the present study, *Sjpcdp*10 mRNA was expressed in all investigated schistosomula developmental stages, with *Sjpcdp*10 expression level elevated along with schistosomula growth and development (Fig. 2). Similar trends were observed for *Sj*PCDP10 protein expression at different schistosomula stages according to western blot analysis (Additional file 4: Figure S1). This indicated that the demand for *Sj*PCDP10 increased along with continuous schistosomula growth and development, suggesting that *Sj*PCDP10 might be involved in schistosomula development. Immunohistochemical analysis revealed that *Sj*PCDP10 was mainly distributed in the teguments and the parenchymal areas of schistosomula at different developmental time points (Fig. 3). The predominant occurrence of *Sj*PCDP10 in the tegument of schistosomula suggested that *Sj*PCDP10 might be a potential functional molecule on the surface and that its function might be related to the tegument/subtegument.

RNAi has been widely used to study the functions of *S. mansoni* and *S. japonicum* at different growth stages [8, 21–24]. The optimal RNAi conditions for knocking down *Sjpcdp*10 in *S. japonicum* schistosomula were systematically determined according to various parameters, including dsRNA concentrations and soaking periods. Results based on qPCR and western blot revealed that the optimal dsRNA concentration was 4 × 10^− 5^ mg/ml, and the optimal incubation period (soaking time) was 6 days (Fig. 4). Under these conditions, the sizes of treated schistosomula were significantly lower than those in the untreated control group (Table 1). Moreover, the surfaces of treated schistosomula were seriously damaged, with invaginations, interruptions, and fewer spines, as well as sensory papillae on the surface (Fig. 6). Programmed cell death (PCD) is a physiological cell death mechanism that can have a positive effect on organism growth and development. In some cases, PCD is fundamentally important for normal growth, as well as to overcome adverse environments [25, 26]. The tegument of schistosomes represents the first physical line of defence against the host environment and the main site of host-parasite interaction. The tegument of schistosomes functions in areas that include nutrient uptake, immune evasion, excretion, osmotic pressure regulation, and signal transduction [27, 28]. Due to the active growth and metabolism of *S. japonicum*, the tegument is constantly being shed, and damaged teguments must be quickly repaired [29, 30]. Although most schistosomula of each group could live well in the RNAi experiment (Additional file 6: Figure S3), serious damage was observed in the teguments of schistosomula following *Sjpcdp*10 knockdown (Fig. 6), suggesting that *Sj*PCDP10 might affect tegument development.

In humans, PCDP10 is an apoptosis-related gene [25]. When PCDP10 was added to the cell culture medium of a human embryonic kidney cell line 293, the number of apoptotic cells decreased along with increasing protein dosage [12]. Moreover, in some studies involving increased apoptosis induced by certain diseases, PCDP10 expression was also upregulated to inhibit apoptosis [31, 32]. In this study, the rate of apoptosis in the *Sjpcdp*10*-*knockdown group increased significantly as compared with that in the blank control group (Fig. 7), which corresponded to the reported role of *Homo sapiens* PCDP10. This suggested that *Sj*PCDP10 might be involved in *S. japonicum* growth and development, as well as apoptosis-related processes, which include tegumental repair and turnover processes.

## Conclusions

In summary, the findings presented here demonstrated that *Sj*PCDP10 was abundantly expressed in the schistosomulum tegument and parenchymal areas, and knockdown experiments showed that *Sj*PCDP10 affected schistosomula growth and development, especially the development of the tegument. The mechanism by which *Sj*PCDP10 influences schistosomula growth and development might involve the regulation of cell apoptosis. Our findings showed that *Sj*PCDP10 plays an important role in schistosomula growth and development.

## Additional files

Additional file 1: Table S1. Primer sequences for real-time qPCR. (XLSX 10 kb)

Additional file 2: Table S2. Primer sequences for preparing dsRNA templates. Underlined sequences indicate the T7 promoter recognition site. (XLSX 9 kb)

Additional file 3: Table S3. Semi-quantitative analysis of *Sj*PCDP10 through the results of immunofluorescence. Stages included skin-stage schistosomula (30 min), 3-day-old lung-stage schistosomula (3 d), 10-day-old liver-stage schistosomula (10 d), 14-day-old liver-stage schistosomula (14 d), 18-day-old liver-stage schistosomula (18 d), and 21-day-old liver-stage schistosomula (21 d). (XLSX 10 kb)

Additional file 4: Figure S1. Western blot analysis of *Sj*PCDP10 protein expression at different time points of the schistosomulum stage of the *S. japonicum* life-cycle. Stages included skin-stage schistosomula (30 min), 3-day-old lung-stage schistosomula (3 d), 10-day-old liver-stage schistosomula (10 d), 14-day-old liver-stage schistosomula (14 d), 18-day-old liver-stage schistosomula (18 d), and 21-day-old liver-stage schistosomula (21 d). The expression of *S. japonicum* α-tubulin was used as an internal control. (TIFF 157 kb)

Additional file 5: Figure S2. Thickness measurement of the schistosomula integument by IPP6.0 software after *Sjpcdp*10-knockdown. Control: schistosomula from the blank control group; EGFP: schistosomula from the *egfp* negative control group; *Sj*PCDP10: schistosomula from the *Sjpcdp*10-knockdown group. (TIFF 28 kb)

Additional file 6: Figure S3. The vitality detection of schistosomulae after *Sjpcdp*10-knockdown at the optimal RNAi optimal condition. **a** the schistosomula of the blank control group; **b** the schistosomula of the *egfp* negative control group; **c** the schistosomula of the *Sjpcdp*10-knockdown group. Most schistosomula of each group could live well in the RNAi experiment. (TIFF 7854 kb)

## Abbreviations

cDNA: Complementary DNA
EGFP: Enhanced green fluorescent protein
PCD: Programmed cell death
PCDP10: PCD protein 10
PCR: Polymerase chain reaction
qPCR: Quantitative real-time PCR
RNAi: RNA interference
r*Sj*PCDP10: Recombinant *Sj*PCDP10
RT-PCR: Reverse transcription PCR
SEM: Scanning electron microscopy
*Sjpcdp*10: *S. japonicum pcdp*10
TBS/T: Tris-buffered saline with 0.05% (*v*/v) Tween-20
TEM: Transmission electron microscopy
TUNEL: Terminal deoxynucleotidyl transferase dUTP nick-end labeling
