# Supplementary figures and images for: Influence of Schistosoma japonicum programmed cell death protein 10 on the growth and development of schistosomula
Source: Parasit Vectors. 2018 Jan 18;11:46. doi: 10.1186/s13071-018-2636-8 (PMC5774102; doi:10.1186/s13071-018-2636-8)

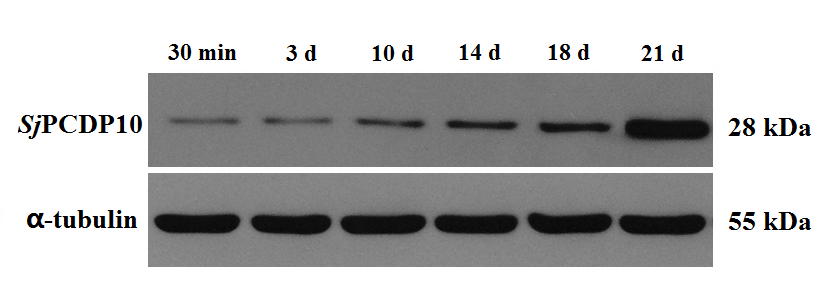

Supplement: Supplementary file 4 — Western blot analysis of SjPCDP10 protein expression at different time points of the schistosomulum stage of the S. japonicum life-cycle. Stages included skin-stage schistosomula (30 min), 3-day-old lung-stage schistosomula (3 d), 10-day-old liver-stage schistosomula (10 d), 14-day-old liver-stage schistosomula (14 d), 18-day-old liver-stage schistosomula (18 d), and 21-day-old liver-stage schistosomula (21 d). The expression of S. japonicum α-tubulin was used as an internal control. (TIFF 157 kb) [file 13071_2018_2636_MOESM4_ESM.tif]

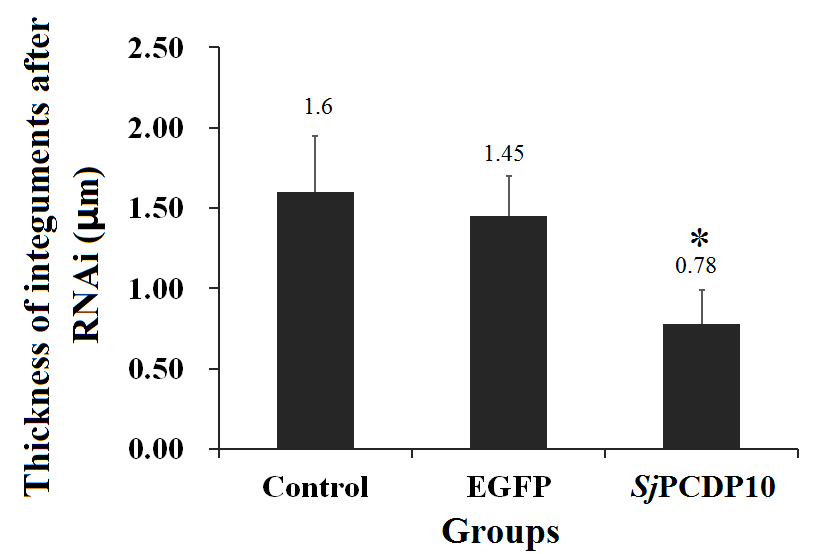

Supplement: Supplementary file 5 — Thickness measurement of the schistosomula integument by IPP6.0 software after Sjpcdp10-knockdown. Control: schistosomula from the blank control group; EGFP: schistosomula from the egfp negative control group; SjPCDP10: schistosomula from the Sjpcdp10-knockdown group. (TIFF 28 kb) [file 13071_2018_2636_MOESM5_ESM.tif]

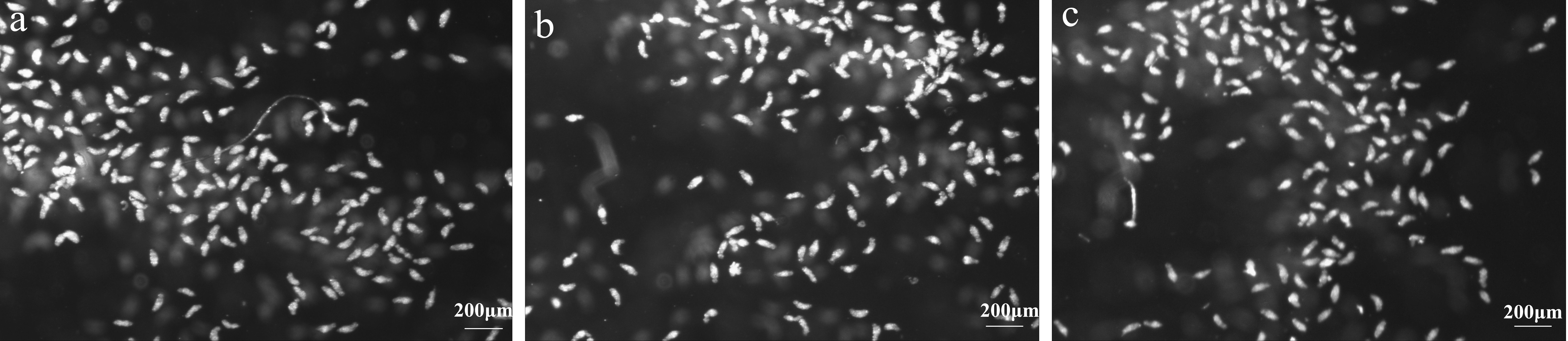

Supplement: Supplementary file 6 — The vitality detection of schistosomulae after Sjpcdp10-knockdown at the optimal RNAi optimal condition. a the schistosomula of the blank control group; b the schistosomula of the egfp negative control group; c the schistosomula of the Sjpcdp10-knockdown group. Most schistosomula of each group could live well in the RNAi experiment. (TIFF 7854 kb) [file 13071_2018_2636_MOESM6_ESM.tif]
